# Introduction: Mnemonic Solidarity—Global Interventions

**DOI:** 10.1007/978-3-030-57669-1_1

**Published:** 2021-02-11

**Authors:** Jie-Hyun Lim, Eve Rosenhaft

**Affiliations:** 3 Department of History and the Critical Global Studies Institute, Sogang University, Seoul, Korea (Republic of); 4 School of Histories, Languages & Cultures, University of Liverpool, Liverpool, UK; 5 Department of History and the Critical Global Studies Institute, Sogang University, Seoul, Republic of Korea; 6 School of Histories, Languages & Cultures, University of Liverpool, Liverpool, UK

**Keywords:** Global memory formation, Mnemonic solidarity, Global South, Territorialization, Local memory

## Abstract

Lim and Rosenhaft introduce “mnemonic solidarity” as a scholarly and political program, situating it in the context of the wider project and publication series “Entangled Memories in the Global South.” Their programmatic approach arises from the observation that a global memory formation has emerged since the late twentieth century, involving interchanges of various kinds between national memory cultures and structured by the terms of Holocaust memory. This development and its political implications have been addressed in various ways by scholars under the rubrics of “cosmopolitan,” “multidirectional,” “traveling,” “prosthetic,” “transnational,” and “agonistic” memory, but the new field of memory studies remains Eurocentric and relatively insensitive to the double-edged character of globalized memory—the interplay between de-territorialization and re-territorialization. This volume aims to reset the agenda.

**Fig. 1.1:**
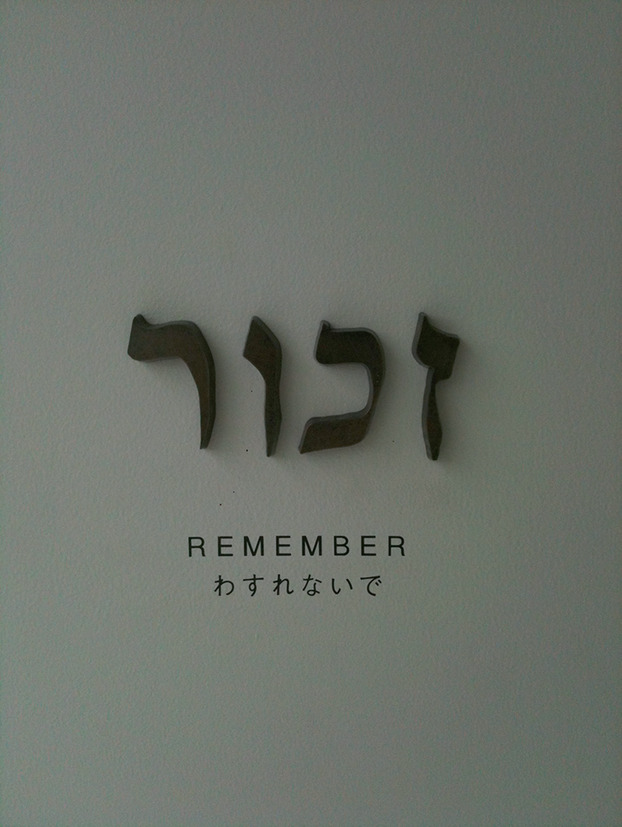
The tablet at the entrance to the Holocaust Education Center in Fukuyama, Hiroshima Prefecture, which is dedicated to Anne Frank’s legacy, August 2012 (Jie-Hyun Lim)

This volume introduces a new publication series and a new emphasis in memory studies. The title of the series is *Entangled Memories in the Global South*. The term “mnemonic solidarity” which gives this book its title signals one response to the observation that historical memories have become entangled. It proposes that that entanglement invites us to rethink memory studies as a field of scholarship and also the sociocultural and political practices through which communities engage with their respective and shared histories. The central question implicit in the term “mnemonic solidarity” is how and how far it is possible to find a common ground for articulating the hurts of the past in ways that are productive for the future. The question itself is not a new one; it has been posed and answered in decades of practical and theoretical work on projects for transitional, commemorative, compensatory and restorative justice, and for historical reconciliation in particular conflict zones. The question takes on new dimensions with the emergence of the global memory formation: Historical experiences are being articulated as memory not only through interactions among the subjects of those histories but also in conversation with the historical memories of others around the world. And in those conversations the lives and voices of historical actors in the global South are increasingly heard in their own terms.

Here, we need to clarify our use of the term “global South.” It does not in any sense represent a geo-positivist fixation, although it corresponds largely to the tri-continent: Asia, Africa, and Latin America. In the series, we use “North” and “South” as liquid geo-positions and historical constructs depending on the ways in which, at a given historical moment, events, questions, and actors are discursively located in global interactions.^1^ At the founding conference of the Non-Alliance Movement in Bandung in 1955, for example, Japan and China belonged to the global South; this is no longer the case. Global interventions expressing mnemonic solidarity between interwar African Americans and Japanese Americans self-defining as Pacific Negroes, between the Irish in the potato famine and the Choctaw native nation after the “Trail of Tears,” between Hiroshima and Auschwitz, between Muslim women victims of sexual violence in former Yugoslavia and East Asian comfort women witnessing in the transpacific space have taken place in the Northern hemisphere. But we include these interactions among “entangled memories in the Global South” because they represent suppressed voices and memories which have become to be heard with the emergence of the global memory formation. The essays in this volume explore the dimensions and implications of that global memory formation from a variety of disciplinary and regional perspectives. The authors, representing two generations of scholars, base their reflections on their study of particular histories and memory formations and also on experiences of active engagement in public history and commemoration.

The global memory formation of which we speak reflects the ways in which globalization has dramatically reconfigured the landscape of memory in the third millennium. The space in which collective memories take shape is no longer national but global, and memories have become entangled, reconciled, contested, conflicted, and negotiated across borders, connecting historical actors and events across time and space. “Formation” needs to be understood in this context as process rather than structure, and the process was accelerated (if not set in motion) by a particular historical moment: As Jie-Hyun Lim shows in his chapter, it was the thaw of memories that had been frozen under the restraint of Cold War ideologies that accelerated this global memory formation and gave new impetus to rewritings of the past, as suppressed memories of the Stalinist terror and Nazi collaboration in Eastern Europe joined new articulations of colonial trauma in the tri-continent of Africa, Asia, and Latin America.

Memory is the posthumous history of history, where interventions are constantly made to rearticulate what happened in the past. In these terms, the emerging global memory formation has two defining features. The first is a complex interplay between de-territorialization and re-territorialization. Across the globe, vernacular and institutionalized memories of past traumas are being shaped in conversations both within and across national, regional, and continental borders. Collective memories shaped in specific local, regional, or national contexts have become interwoven with one another through processes and practices of translation, cross-referencing, adaptive imagination, unilateral “re-purposing” and active dialogue, as well as competition. Almost without exception, the global South tends to create its own mnemoscape through the dynamics of comparison, cross-referencing, juxtaposition to and repulsion from the Holocaust in the global North. Many memory activists in the global South have adopted these practices as a deliberate tactic for marking out their own position in the global memory formation. And in fact testimony to and memories of human rights abuses in the global South have attracted the attention of the global public sphere largely when and as they became more interactive or entangled with the Holocaust as the ethical norm of memories. There is a degree of randomness in the way in which the remembrance of transatlantic slavery, the Nanjing massacre, the atomic bombing of Hiroshima and Nagasaki, and even the comfort women have adopted the language of Holocaust. But as Eve Rosenhaft’s analysis of Black Holocaust fictions proposes, that discursive nexus has an imaginative power that reflects an authentic de-territorialization—of the tools or terms of memory, at least.

At the same time, though, the global memory formation has contributed to re-territorializing the mnemoscape by providing a new frame for heightened competition among the parties to contending national memories. Perhaps the best example of this is the way in which the globalization of Holocaust discourse has been accompanied by its appropriation in political conflicts within and between nation-states. The results of such juxtapositions can be simply scandalous. In Eastern Europe post-Communist states have nationalized Holocaust remembrance to justify a resurgent old-fashioned ethnic nationalism and provide a screen memory that obscures their own war crimes. Even among Europeans for whom the Second World War and the Holocaust are, after all, part of their local memory, the dimensions of the claims to victimhood that can be made in terms of Holocaust are practically kaleidoscopic, showing new complications as each national trauma enters into the conversation. It is disturbing, for example, to witness the efforts of the “Jasenovac Committee of the Synod of Bishops of the Serbian Orthodox Church” since the end of the Balkan civil wars to rehabilitate the Serbian-Chetnik fascists as concentration camp victims—in close collaboration with the World Holocaust Remembrance Center at Yad Vashem.^2^

This is the first ground on which the project represented by this volume responds to established currents in memory studies: The perception that key components of cultural memory—identificatory narratives about the past generated in one place or by one mnemonic community^3^—can be and have been appropriated by cultural and political actors outside that community has generated some key terms in the developing field of memory studies. At the beginning of the twenty-first century, Daniel Levy and Natan Sznaider identified a formation of “cosmopolitan memory” in the global circulation of Holocaust discourse.^4^ In 2009, Michael Rothberg introduced the term “multidirectional memory” to characterize the overlaps and exchanges between Holocaust and (post)colonial memory.^5^ Astrid Erll’s reflections on the future of memory studies in the light of the manifest porousness of the nation-state “container” led her by way of “transcultural memory” to the influential coinage “traveling memory.”^6^ And the concept of “prosthetic memory” proposed by Alison Landsberg was essentially an answer to the question of how members of one mnemonic community can internalize the “memories” of another. Although it emerged from a study of American memory cultures, Landsberg’s proposition rested on observations about global transformations in the conditions for memory, notably in the technologies through which experience is communicated.^7^ The investigation of these dynamics has also been carried out under the rubric “transnational memory.”^8^

The mnemonic solidarity project builds on the insights and methods of all of those scholars, but it starts from an acute awareness that the global memory space is a double-edged formation which promotes the de-territorialization and re-territorialization of remembrance simultaneously. Our concern is less with the traces of cosmopolitan memory than with continuing challenges to productive interchange between communities of memory. The forms of selective remembering that we call re-territorialization need to be anatomized and critiqued before we can move on to construct genuinely usable narratives of the pasts we share. These developments call for a program of critical rethinking which is both scholarly and political: How have particular memories and memory practices emerged out of particular historical experiences, how have they come to be appropriated as official or cultural memory or for deployment in civil and international conflicts, and what specific role does transnational exchange—the entanglement of memories—play in the formation of memory and memory practices?

Those earlier models took as their starting point questions of Holocaust memory. Reflections on how, where, when, and by whom that epochal moment in European history has been remembered have been foundational for the field of memory studies since the 1970s. Mainstream studies have built on theoretical foundations laid in the European sociological tradition and persistently focused on the European and American experiences.^9^ This leads us to the second feature of the new global memory formation that this volume addresses and which is at the core of its underlying rationale: Even as the pull of the European ( Holocaust) experience continues to be powerful in global articulations of trauma, that experience is being increasingly de-centered. That same post-Cold War thaw that released suppressed memories of Stalinist terror and Nazi collaboration in Eastern Europe presaged new articulations of the violence of colonialism and neo-imperialism in other parts of the world. The rhythms and outcomes of these articulations were not uniform. In Latin America’s Southern Cone, for example, the 1990s democratization was followed by an initial closing down of public discussion of Pinochet’s dictatorship in Chile, while Argentina, whose dictatorship had ended a decade earlier, experienced a generational shift from the preoccupation with justice to concerns with memory.^10^ In general, though, memorial practices and the critical study of them have increasingly partaken of international conversations in which scholars and activists from the global South have taken a lead. New work in this area reflects on the utility and capacity of both new technologies and repurposed everyday practices to articulate identities and empower activists at the regional level, exploring the paradoxes of re-territorialization through transnational media.^11^

This opening up has also led to new, non-hierarchical appreciations of the comparability of historical traumas. The Holocaust is ceasing to be the model of which other traumas were versions, and has become subject to postcolonial readings itself. These locate the Holocaust in the history of global colonialism, elaborating its place in a continuous development beginning (for Germany) with genocidal campaigns in German Africa and situating the German invasion and occupation of the Slavic East firmly in the European colonialist tradition. In Chap. 10.1007/978-3-030-57669-1_2, Jie-Hyun Lim elaborates this re-visioning of the Holocaust and some of the ways in which this new formation is manifested in public discourse.

Equally significant is the audibility of new actors—the global South—in the global memory formation. As Eve Rosenhaft proposes in her anatomy of “Europe’s melancholias,” people voicing the colonial and postcolonial experience from positions *within* the global North are now part of conversations about how past and present connect. Their perspectives on the Holocaust and its lessons, brought into contention with received narratives in a moment of political crisis, mark a mnemonic moment which is arguably as much post-Holocaust and post-postwar^12^ as it is postcolonial and (surprisingly) postimperial. Global perspectives open up new temporalities, which in turn make us newly attentive to what has been forgotten or suppressed in the construction of memories.

Carol Gluck’s meticulous account of how the East Asian comfort woman came to be a new global icon for historical trauma and accountability draws together key elements of the global memory formation. It exposes the importance of particular conjunctures—temporal moments—in the public understanding and speakability of human rights and war crimes. Central to the story is, of course, the global visibility of the East Asian experience of war and the entry of East Asian (women) actors into transnational mnemonic conversations. As Jie-Hyun Lim also intimates, memory developments in East Asia are in important ways fundamental to the global memory formation. Not only has the western Pacific rim been the site of intense memory conflicts arising out of the complex imperial, colonial, and postcolonial relationships among China, Japan, and Korea, but patterns of memory politics there have been very much informed by discourses of victimhood and responsibility that originated in the West. At any rate, this is how things look through the lens of Western scholarship.

If the first three substantive chapters in this volume map out some fairly familiar territory in the global mnemonic landscape, then, they also point in the direction of new themes and questions that are foundational for the mnemonic solidarity project. One of these is how the field of memory studies itself may change as experiential perspectives and scholarly voices from Africa, Asia, and Latin America enter into the discussion—or, more radically, when we take them as our starting point. A first step here will be to take them seriously in their own terms, articulating, for example, what distinguishes East Asian memory regimes and the preconditions for memory practices *as well as* how they have appropriated Western models of “memory contest.” For example, Carol Gluck reminds us that “the geopolitical postwar era in East Asia and Eastern Europe really began only after 1989.”^13^

In Chap. 10.1007/978-3-030-57669-1_5, Lauren van der Rede and Aidan Erasmus invite us to take “Africa” on its own terms. Mainstream memory studies that focus on Africa have begun with institutions and practices prompted by interventions from diasporic and international agencies (the memorialization of transatlantic slavery), literary mediations in forms marketable to European and American audiences, or post-conflict and post-genocide issues of justice and representation drawing on international models and comparisons.^14^ (A notable exception here is South Africa, where the injustices of apartheid were the object of global interventions before they became the subject of memory and both scholars and activists have historically operated transnationally—often representing the global South in the global North and vice versa.) Examining the cases of Ethiopia and South Africa, van der Rede and Erasmus provocatively characterize Africa as a “disobedient object” of memory studies, posing a series of radical challenges to the terms and methods of the field. At the empirical level, they point out how these cases inflect our Europe-centered models of trauma and memory. In the Ethiopian context, the forensic vocabulary introduced by post-Holocaust human rights law and discourse have been redefined in the legislative negotiation between “genocide” and “terror.” In South Africa, the institutions and mentality that underpinned the apartheid system can be seen as resting in turn on a mnemonic infrastructure in which colonial hybridity and the identity of a nation in arms were entangled in very particular ways. Beyond this, positing Africa “not as a cartographic and geological location but as a concept and methodology,” van der Rede and Erasmus challenge the liberal universalism implicit in the problematics of memory studies (and indeed in the notion of mnemonic solidarity) with an insistence on hearing/listening rather than speaking that draws on postcolonial theory and the new methods of sound studies. Mnemonic solidarity retains more than heuristic power as a normative real, but it is precisely the ways in which the de-centering of global North perspectives tests it to its limit that constitute the intellectual promise of a genuinely globalized memory studies.

One thing that is at issue in van der Rede and Erasmus’ critique of liberal universalism is the obligation to speak which the emphasis on witnessing in Holocaust-informed memory studies places on the subjects of memory. This addresses the second key move in the mnemonic solidarity project: critical attention to specific actors and material processes. Who are the rememberers and what are they able to say? In memory studies as in other disciplines that employ the language of globalization, there is a danger that “territorialization” and its variants come to denote disembodied forces.^15^ Our model of global memory formation is a dynamic one; far from being a simple piling-up of individual national memories, it regulates and stimulates national remembrance by co-figuring national memories—most obviously, in the self- and other-identities of perpetrator and victim nations. That formation depends in turn on the internal dynamics of national and local memory communities. Even if we fix our attention at the level of the national, the analysis of re-territorialization needs to take into account the mechanisms through which official memory regimes selectively appropriate, pre-empt and silence vernacular memory. But of course there are contests, too, among and within “grass-roots” memory communities, most acute among survivors of political repression and genocide. And memory communities themselves are subject to being reshaped and fractured through temporal processes of generational and demographic change, such as Eve Rosenhaft explores in Chap. 10.1007/978-3-030-57669-1_3.

This calls for caution. Acknowledging the agency and eliciting the voices of subaltern and marginalized historical actors, irrespective of where they were positioned in moments of historical trauma (whether as “victims,” “perpetrators,” or “bystanders”), are essential to the democratization of both narratives and resources that is part of the mnemonic solidarity project. But we need to be alert to ambivalences at the vernacular level, too. Speech may prove pointless and dialogue incapable of generating solidarity.^16^ The tendency of the global memory formation to enable conversations between local memory communities is apparent in new forms of transnational memory activism, like the multiple border-crossings of the South Korean comfort woman statue discussed here in the chapters by Jie-Hyun Lim and Carol Gluck. But the popularization of national victimhood narratives and the mobilization of grass-roots actors to defend them in acts of performative nationalism, such as we see in the case of the comfort women, bespeaks the double-edged quality of memory formation at this level.^17^

Attention to the possibilities for making memory “from below” raises the question of what tools the memory makers have available: the material, institutional, and cultural conditions for the construction of vernacular memories and their articulation in and with national and global conversations. These questions are sometimes answered by giving attention to actors and events at the very local level, and this is a frontier of research whose importance we want to signal although it is not represented elsewhere in the present volume. On the one hand, locality itself is an important determinant of identity and an object of memory. The neighborhood around the Bataclan nightclub in Paris, site of a terrorist attack in November 2015, and the South Korean city of Gwangju, subject to violent repression of a democracy movement in 1980, provide examples of the power of local memory, though with notable differences.^18^ In the case of cities, what is remembered locally is often the struggle to retain the physical fabric of memory itself: the visible traces of a community. This is well represented in protests against redevelopment which articulate the nexus between identity and the configuration of urban space—examples of what Edward S. Casey calls “place memory” and of Andreas Huyssen’s “urban imaginary.” These forms of memory are haunted by the global, as resistance has often adopted the voice of nostalgia for neighborhood pasts characterized by cosmopolitan values and ethnic and social diversity.^19^

And there are other ways in which “glocal,” that coinage of the 1990s, is relevant to questions of memory and mnemonic solidarity. Where most of the contributions to this volume refer to the traumas of war and genocide, “rebel cities” typically articulate the material and psychological traumas incurred by neoliberalism at the intersection of aesthetics and everyday life—where the city itself is a victim of global capital flows that drive the privatization and homogenization of urban space.^20^ Per contra, in the form of housing activism, urban memory movements have acquired global networks and vocabularies.^21^ It is also the case that some icons of trauma which circulate globally have very particular associations for the memory communities in the places where the event took place—associations shaped by pre-existing discourses of local identity. An example of this is the 2001 attack on the World Trade Center, whose identificatory power and mnemonic complexities for New Yorkers are being evoked by the city’s experience of the coronavirus pandemic as this volume goes to press.^22^ It is at the local level, too, that insurgent memories arise out of everyday hurts. Formulated as demands for justice that expose structural inequalities in democratic societies and reinforced in commemorations that enact counter-national identities, these, too, can now go global. Here, the editors of this volume cannot fail to mention the solidary encounters between the Liverpool families of the victims of the 1989 Hillsborough Disaster and those of the people (mainly teenagers) who drowned in the sinking of the Sewol Ferry off the South Korean coast in 2014.^23^ Theirs are also voices of a global South.

## Footnotes

1 See Arif Dirlik, “Global South: Predicament and Promise,” *The Global South* 1, no. 1 (2007): 12–23; Anne Garland Mahler, “Beyond the Color Curtain. The Metonymic Color Politics of the Tricontinental and the (New) Global South,” in *The Global South Atlantic*, eds. Kerry Bystrom and Joseph R. Slaughter (New York: Fordham University Press, 2018), 99–123.

2 Jovan Byford, “When I Say ‘the Holocaust’ I Mean ‘Jasenovac’: Remembrance of the Holocaust in contemporary Serbia,” *East European Jewish Affairs* 37, no. 1 (2007): 51–74.

3 The earliest deployment of the term is by Eliatar Zerubavel, “Social Memories: Steps to a Sociology of the Past,” *Qualitative Sociology* 19, no. 3 (1996): 283–99 (here 289–91).

4 Daniel Levy and Natan Sznaider, “Memory Unbound: The Holocaust and the Formation of Cosmopolitan Memory,” *European Journal of Social Theory* 5, no. 1 (2002): 87–106; Idem., *The Holocaust and Memory in the Global Age*, trans. Assenka Oksilloff (Philadelphia: Temple University Press, 2006).

5 Michael Rothberg, *Multidirectional Memory: Remembering the Holocaust in the Age of Decolonization* (Stanford: Stanford University Press, 2009).

6 Astrid Erll, “Travelling Memory,” *Parallax* 17, no. 4 (2011): 4–18.

7 Alison Landsberg, *Prosthetic Memory. The Transformation of American Remembrance in the Age of Mass Culture* (New York: Columbia University Press, 2004).

8 Chiara de Cesari and Ann Rigney, eds., *Transnational Memory: Circulation, Articulation, Scales* (Berlin and Boston: de Gruyter, 2014).

9 Reference here is to the work of Maurice Halbwachs in the 1920s, as critiqued and elaborated by Jan and Aleida Assmann in the 1990s: Maurice Halbwachs, *On Collective Memory*, ed. and trans. Lewis A. Coser (Chicago: University of Chicago Press, 1992); Jan Assmann, “Collective Memory and Cultural Identity,” *New German Critique* 65 (1995): 125–33; Aleida Assmann, *Cultural Memory and Western Civilization: Functions, Media, Archives* (Cambridge: Cambridge University Press, 2011) (originally published in German in 1999). For a recent critique of the Western-centrism of memory studies, see Hunmi Lee et al., “Conference Report: The Third Annual Conference of the Memory Studies Association in Madrid, 2019,” accessed November 11, 2020, http://cgsi.ac/bbs/board.php?bo_table=eng_e_Pub&wr_id=6.

10 Eugenia Allier Montaño and Emilio A. Crenzel, eds., *The Struggle for Memory in Latin America. Recent History and Political Violence* (Basingstoke: Palgrave Macmillan, 2015).

11 Claire Taylor and Thea Pitman, “Conclusion: Latin American Identity and Cyberspace,” in *Latin American Cybercultures and Cyberliterature*, eds. Claire Taylor and Thea Pitman (Liverpool: Liverpool University Press, 2007), 263–67; Claire Taylor, *Place and Politics in Latin American Digital Culture. Location and Latin American Net Art* (New York: Routledge, 2014); Tania Pérez-Bustos, Eliana Sánchez-Aldana and Alexandra Chocontá-Piraquive, “Textile Material Metaphors to Describe Feminist Textile Activisms: From Threading Yarn, to Knitting, to Weaving Politics,” *TEXTILE* 17, no. 4 (2019): 368–77; Thi Ry Duong, Edward Little and Steven High, eds., *Remembering Mass Violence: Oral History, New Media and Performance* (Toronto: University of Toronto Press, 2014). We regret that it was not possible to include a contribution from Latin America in this volume.

12 In the light of the developments explored in this volume, there is some irony in the observation with which Tony Judt introduced the coda to his 2005 study of Europe’s successful reconstruction based on “selective forgetting”: “As Europe prepares to leave World War Two behind … the recovered memory of Europe’s dead Jews has become the very definition and guarantee of the continent’s restored humanity.” Tony Judt, *Postwar. A History of Europe since 1945* (London: Heinemann, 2005), 804.

13 See also Sheila Miyoshi Jager and Rana Mitter, “Introduction: Re-Envisioning Asia Past and Present,” in *Ruptured Histories: War, Memory, and the Post-Cold War in Asia*, eds. Sheila Miyoshi Jager and Rana Mitter (Cambridge MA: Harvard University Press, 2007), 1–14. Questions of the regional particularities of the East Asian memory wars and possible approaches to reconciliation are also explored in *Northeast Asia’s Difficult Past. Essays in Collective Memory*, eds. Mikyoung Kim and Barry Schwartz (Basingstoke: Palgrave Macmillan, 2010) and *East Asia beyond the History Wars. Confronting the Ghosts of Violence*, eds. Tessa Morris-Suzuki et al. (Oxford and New York: Routledge, 2013).

14 Marie-Aude Fouéréa and Lotte Hughes, “Heritage and memory in East Africa today: a review of recent developments in cultural heritage research and memory studies,” *Azania: Archaeological Research in Africa* 50 (2015): 542–58; Erica L. Johnson and Éloïse Brezault, eds., *Memory as Colonial Capital. Cross-Cultural Encounters in French and English* (Cham: Palgrave Macmillan, 2017).

15 See, for example, the critique by Stef Jansen and Staffan Löfving cited by Chiara de Cesari and Ann Rigney in their introduction to *Transnational Memory* (1–25, here 13), and the emphasis that that volume places on concrete, actor-led processes of articulation and circulation and the conditioning element of “scale.”

16 In memory studies, the power of dialogic confrontation between contending memories is being explored in discussions of “agonistic memory”: Anna Cento Bull and Hans Lauge Hansen, “On Agonistic Memory,” *Memory Studies* 9, no. 4 (2016): 390–404; Cristian Cercel, “The Military History Museum in Dresden: Between Forum and Temple,” *History & Memory* 30, no. 1 (2018): 3–39.

17 On the performative as a negotiation of popular and institutional visions of nation, see Homi Babha, “DissemiNation: Time, narrative and the margins of the modern nation,” in idem., *The Location of Culture* (London: Routledge, 1994), 199–244 (here 210–17). See also Jie-Hyun Lim, “Transnational Memory Activism and Performative Nationalism,” in *Handbook of Memory Activism*, eds. Jenny Wüstenberg et al. (forthcoming 2021).

18 Sarah Gensburger, *Memory on My Doorstep: Chronicles of the Bataclan Neighborhood, Paris 2015–2016*, trans. Katharine Throssell (Leuven: Leuven University Press, 2019); Linda S. Lewis, *Laying Claim to the Memory of May: A Look Back at the 1980 Kwangju Uprising* (Honolulu: University of Hawai’i Press, 2002), especially 135–51 on the simultaneous consolidation of a local identity around the trauma and efforts to insert the commemoration in the global human rights program.

19 Edward S. Casey, *Remembering. A Phenomenological Study* (Bloomington: Indiana University Press, 2000), 181–215; Andreas Huyssen, “Introduction,” in *Other Cities, Other Worlds*, ed. Andreas Huyssen (Durham NC: Duke University Press, 2008), 1–23; Dolores Hayden, *The Power of Place: Urban Landscapes as Public History* (Cambridge MA: MIT Press, 1995), 44–81; Yuan Yao and Rongbin Han, “Challenging, but not Trouble-Making: cultural elites in China’s urban heritage preservation,” *Journal of Contemporary China* 25, no. 98 (2016): 292–306; Martin Zebracki, “Urban preservation and the queerying spaces of (un)remembering: Memorial landscapes of the Miami Beach art deco historic district,” *Urban Studies* 55, no. 10 (2018): 2261–85.

20 Cf David Harvey, *Rebel Cities. From the Right to the City to the Urban Revolution* (London: Verso, 2012).

21 Katia Valenzuela-Fuentes, Dominika V. Polanska and Anne Kaun, “The right to housing in theory and in practice: going beyond the West,” *Interface* 9, no. 1 (2017): 359–67.

22 Setha M. Low, “The Memorialization of September 11: Dominant and Local Discourses on the Rebuilding of the World Trade Center Site,” *American Ethnologist* 31, no. 3 (2004): 326*–*39; Simon Stow, “From Upper Canal to Lower Manhattan: Memorialization and the Politics of Loss,” *Perspectives on Politics* 10, no. 3 (2012), 687–700.

23 “Sewol and Hillsborough: families see common threads in tragedy,” *Hankyoreh*, May 16, 2016, http://english.hani.co.kr/arti/english_edition/e_international/743734.html. The meeting was part of a European tour by the Sewol families in which they met with the families of victims of the 1994 sinking of the MS Estonia and of the Paris terror attacks: “Families of Sewol Ferry Victims Meet with Families of Hillsborough and MS Estonia Disaster Victims,” accessed May 14, 2020, https://rememberingsewoluk.files.wordpress.com/2016/05/20160430_pressrelease_engl.pdf.

